# Automatic Polyp Detection in Pillcam Colon 2 Capsule Images and Videos: Preliminary Feasibility Report

**DOI:** 10.1155/2011/182435

**Published:** 2011-05-22

**Authors:** Pedro N. Figueiredo, Isabel N. Figueiredo, Surya Prasath, Richard Tsai

**Affiliations:** ^1^Department of Gastroenterology, University Hospital of Coimbra and Faculty of Medicine, University of Coimbra, 3000-075 Coimbra, Portugal; ^2^CMUC, Department of Mathematics, University of Coimbra, 3001-454 Coimbra, Portugal; ^3^Department of Mathematics, The University of Texas at Austin, Austin, TX 78712, USA

## Abstract

*Background*. The aim of this work is to present an automatic colorectal polyp detection scheme for capsule endoscopy. *Methods*. PillCam COLON2 capsule-based images and videos were used in our study. The database consists of full exam videos from five patients. The algorithm is based on the assumption that the polyps show up as a protrusion in the captured images and is expressed by means of a *P*-value, defined by geometrical features. *Results*. Seventeen PillCam COLON2 capsule videos are included, containing frames with polyps, flat lesions, diverticula, bubbles, and trash liquids. Polyps larger than 1 cm express a *P*-value higher than 2000, and 80% of the polyps show a *P*-value higher than 500. Diverticula, bubbles, trash liquids, and flat lesions were correctly interpreted by the algorithm as nonprotruding images. *Conclusions*. These preliminary results suggest that the proposed geometry-based polyp detection scheme works well, not only by allowing the detection of polyps but also by differentiating them from nonprotruding images found in the films.

## 1. Introduction

Colorectal cancer is a major health problem. In fact, in terms of incidence worldwide, colorectal cancer ranks fourth in frequency in men and third in women, while prevalence is second only to that of breast cancer, mortality being approximately one half that of incidence [1]. Increasing compliance with both initial screening test recommendation and diagnostic testing are cost-effective approaches [2]. 

Colon capsule endoscopy (CCE) may be an alternative strategy to conventional colonoscopy, increasing compliance with screening. The first two papers, comparing CCE with colonoscopy, were published in 2006 and showed that the technique is feasible and safe [3, 4]. Two meta-analysis confirm that CCE is a useful tool for the identification of colonic polyps [5, 6]. Recently, a second version of the colon capsule, with technological improvements, was developed by Given Imaging (Given Imaging, Yoqneam, Israel) [7].

Computer-aided diagnosis, as in CT colonography [8], would be very helpful in CCE by potentially increasing diagnostic performance in the detection of polyps and masses, by decreasing variability of the diagnostic accuracy among readers and, probably, by reducing reading time.

The aim of this paper is to present our approach towards relieving the burden of manually analyzing each of the CCE frames by developing an automatic polyp detection scheme. We utilize the protrusion measure of polyps computed from curvatures of the input image to produce an efficient detection method. In this paper we present some preliminary results of our scheme on a database of PillCam COLON 2 (Given Imaging, Yoqneam, Israel) images and videos.

## 2. Material and Methods

### 2.1. Bowel Preparation and Colon Capsule Endoscopy System

The bowel preparation procedure was similar to the one reported by Eliakim et al. [7] and consisted of a clear diet and a total amount of 4 L of polyethylene glycol (PEG) prior to capsule ingestion, divided in two doses of 2 L, one in the eve and the other in the morning of the examination day. One oral sodium phosphate booster was administered when the capsule entered the small bowel, and another, three hours later, if the capsule had not been excreted by that time.

Briefly, the PillCam COLON 2 measures 11,6 by 31,5 mm, has two imagers, and captures images at a frame rate between 4 images per second, when stationary, and 35 images per second, when in motion. The device is ingested by the patient, which transmits images of the gastrointestinal tract, by an antenna-lead array, to a data-recording device carried by the patient. After the sensor array and the recording device are removed, the digital video image streams of the examinations are downloaded to the RAPID C2 system, and the digital image stream is assessed and interpreted. The capsule procedure was performed without sedation, intubation, or air insufflations.

The capsule was ingested in the morning and conventional colonoscopy, which is the gold standard test, was carried out in the afternoon of the same day, after the excretion of the capsule. 

### 2.2. Capsule Endoscopy Database

The database consists of full exam videos from five patients, three males and two females (mean age 62 years), who underwent CCE with PillCam COLON 2 in our department. They were selected among patients who were referred to our department to undergo colonoscopy. All gave informed consent. 

Seventeen short videos of 100 frames each, containing polyps, flat lesions, diverticula, bubbles, and trash liquids were selected to test the automatic scheme in different scenarios. All the lesions detected by the capsule were confirmed by conventional colonoscopy, and appropriate procedures, including polypectomy and biopsies, were taken according with the endoscopic findings. 

### 2.3. Algorithm Outline

We have devised an automatic image processing algorithm to screen each image frame for potential polyps. Our algorithm is based on the geometric characterization of polyps that appear to be somewhat roundish protrusion from the surrounding mucosal surface. The algorithm can be described in the following steps.

1. A preprocessing step: the input image is clipped around the circular view using a mask (to remove details such as time and patient name), and an automatic illumination correction scheme [9] is applied.
2. We first smooth the illumination corrected image using the heat equation, so that noise in the original images will not affect the polyp detection. Note that this smoothing creates a smooth surface from which polyp-like structures can be inferred using their protrusion amount.
3. The amount of protrusion in the images is gauged by a special function which we call *P*. It is defined based on the curvatures of the image by *P* = −Gc × min (Mc, 0), where Gc = Gaussian curvature and Mc = mean curvature.
4. To further visualize the *P* function we used a circular mask smaller than the original view of the PillCam COLON 2 camera. Mathematically, the value of the function *P* is closely related to the size of the protrusions in the images. Therefore, the polyp location in the frame can be inferred by identifying the locations where *P* is higher.

All the computations were done on MATLAB on a Windows7 machine with Intel Core2 Duo CPU with 3.00 Gb RAM. The main computations were the heat equation computation and the curvature from the smoothed image. For a video with 100 frames at the resolution of 512 × 512 pixels per frame, our first MATLAB implementation of the algorithm takes about two hours, including the visualization of the computation.

## 3. Results

We tested our algorithm in seventeen different short videos of 100 frames each in MPEG format, selectively extracted from the full length capsule exam by using the RAPID C2. 

**Figure 1:**
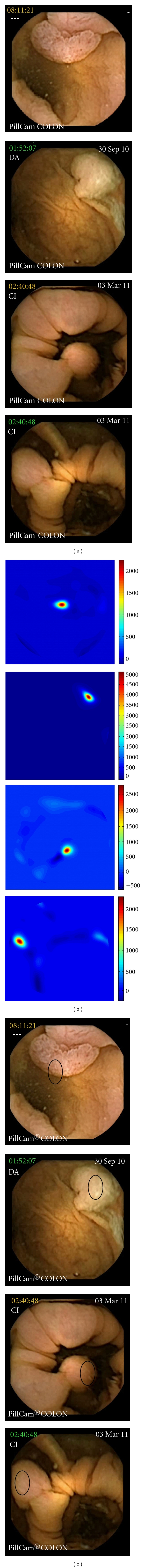
Polyps expressing a *P*-value higher than 2000: (a) original image, (b) computed curvature-based function *P*, (c) the area which falls within the region of the highest *P*-value is shown here using a black contour superimposed on the input image.

**Figure 2:**
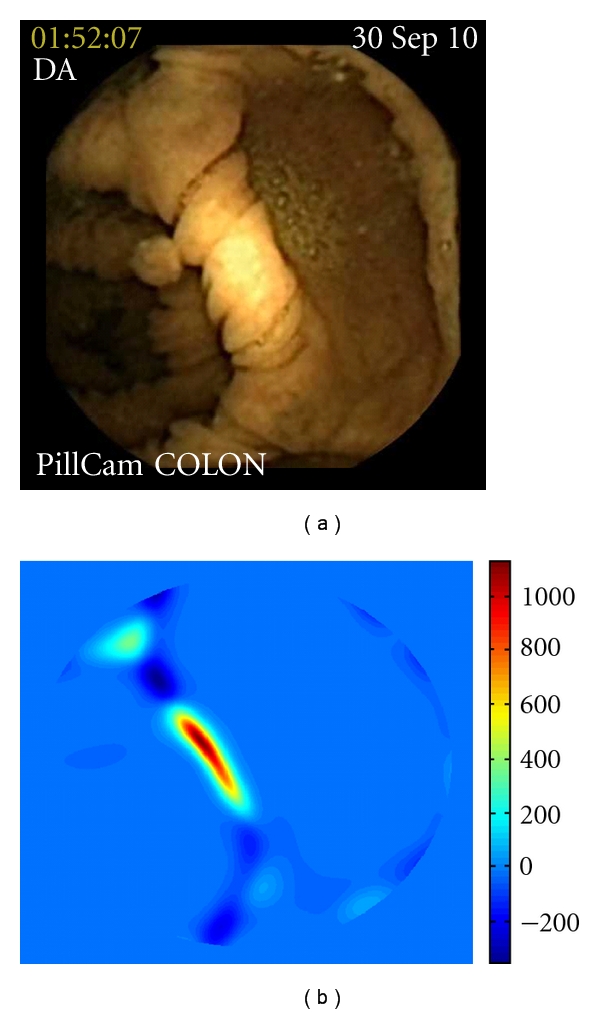
Polyp expressing *P*-value between 1000 and 2000.

**Figure 3:**
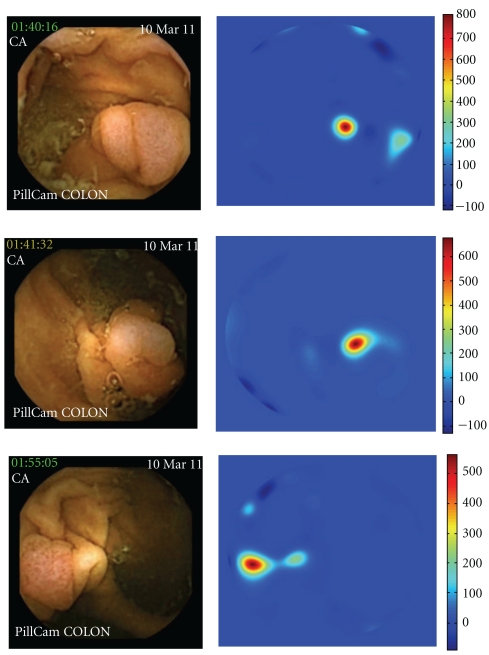
Polyps expressing *P*-value between 500 and 1000.

**Figure 4:**
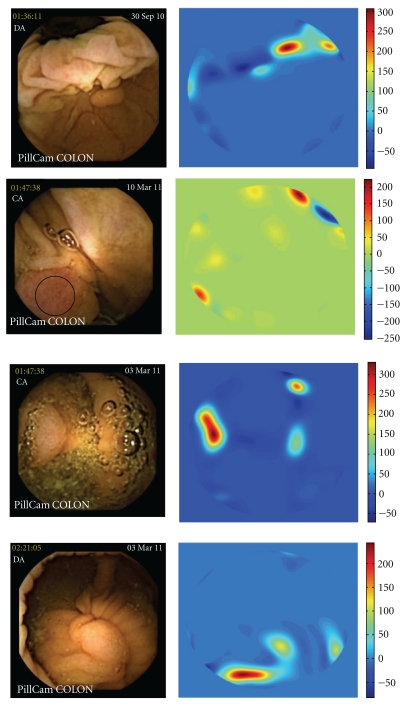
Polyps expressing *P*-value inferior to 500.

Ten polyps were detected in twelve videos (two polyps are seen in two different videos because they were captured by the two imagers, giving a different perspective of the lesion). In four videos, *P*-value was higher than 2000 (Figure 1), in one video *P*-value was between 1000 and 2000 (Figure 2), in three videos *P*-value was between 500 and 1000 (Figure 3), and in four videos *P*-value was inferior to 500 (Figure 4). At conventional colonoscopy, all the polyps were measured. The diameter was higher than 1 cm in the four polyps expressing a *P*-value higher than 2000 and was less than 1 cm when the polyps expressed a *P*-value less than 2000. *P*-value was higher than 500 in 80% of the polyps.

**Figure 5:**
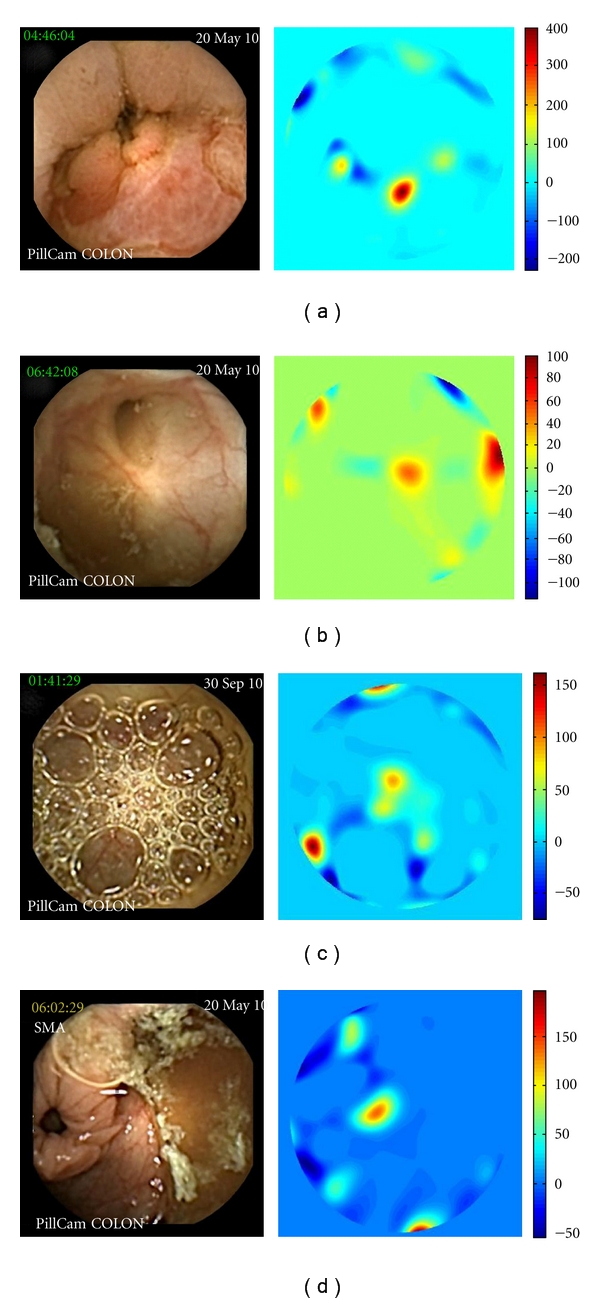
All of the following expressed *P*-value under 500: (a) cecal ulcer, (b) diverticula, (c) bubbles, and (d) trash liquid.

Figure 5 shows the results of the *P* function in frames with a cecal ulcer, diverticula, bubbles, and trash liquid. The *P* function values for these images are very low in each case, always under 500.

**Figure 6:**
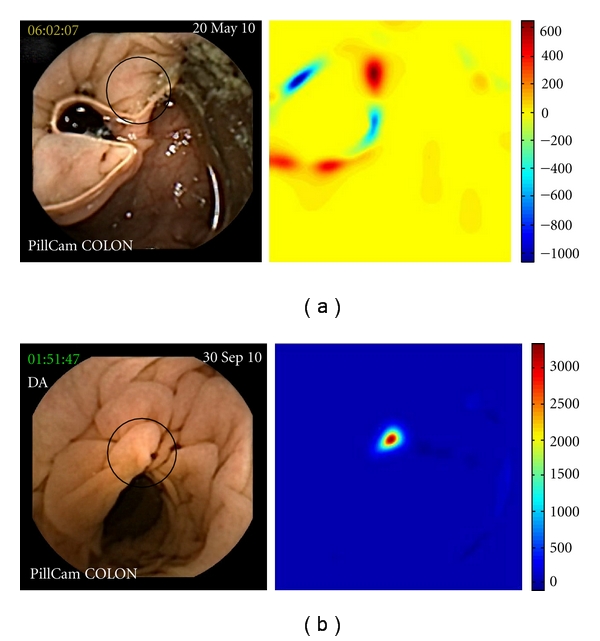
*P*-value of folds (a black contour superimposed on the input image shows the fold recognized by the algorithm).

Finally, Figure 6 shows the result of the *P* function applied to frames with only folds. In Figure 6(a)), which corresponds to a fold highlighted by the capsule, there is some amount of protrusion and hence the range of the computed *P* function, shown in the right of the figure, lies in the range 500 to 1000. In Figure 6(b)), the mucosal fold mimics a polyp, giving a *P*-value higher than 2000. Both results are related with the methodology used in the algorithm, based on the curvatures protruding out of the surrounding mucosal surface

## 4. Discussion

We proposed a geometry-based polyp detection algorithm which has proven to perform well overall. In particular, it correctly detects colonic polyps exhibiting a significant roundish protrusion from the surrounding mucosal surface, showing a *P*-value superior to 2000 when the polyp has a diameter higher than 1 cm, a finding with clinical relevance. Moreover, 80% of the polyps evidence a *P*-value higher than 500, and the detection scheme is clearly robust with respect to trash, bubbles, and diverticula, as illustrated by different examples. 

The main drawback of the proposed approach is the reliance only on the protrusion measure of the polyp to identify potential candidates. The consequence is that if a polyp is not protruding “enough” from the surrounding mucosal folds or a frame does not show the polyp as an indentation among the lumen region, it may be missed by our scheme, resulting in a false negative. Conversely, if a mucosal fold protrudes sufficiently into the lumen, the algorithm shows a high *P*-value, resulting in a false positive. Nevertheless, computer-aided detection systems for CT colonography depending only on the amount of protrusion have been described [10]. 

The main advantage of endoscopy, conventional or capsule based, over CT colonography is that we can rely, not only in protrusion, but also in texture, and probably, in colour. In fact, trying to overcome the limitations of the present algorithm, we are currently investigating a texture-based detection method (coupled with our proposed geometry based methods), aiming to reduce the number of the false negatives and false positives. Another problem is that the computation time is too long, but with an implementation on C language we expect to have at least a five time speed up from our current MATLAB implementation.

In addition, if we can make the scheme “real-time”, then it will be a very useful tool to get an indicator of whether a polyp is present in the current frame or not. This, in turn, will reduce the burden of analyzing all the frames from a full CCE video, saving valuable time.

To the best our knowledge, this is the first paper on automatic detection of colonic polyps recognized by capsule endoscopy. Our preliminary analysis, with a limited number of videos and frames, shows the potential of the polyp detection algorithm proposed in this paper. Given what can be expected from CCE in the future, shortening reading times, assisted by reliable computer algorithms that work as a “pathology indicator”, is obviously a main field of research [11].
